# The role of GPR81–cAMP–PKA pathway in endurance training-induced intramuscular triglyceride accumulation and mitochondrial content changes in rats

**DOI:** 10.1186/s12576-024-00902-x

**Published:** 2024-02-08

**Authors:** Lin Li, Xiangdeng Lai, Yihan Ni, Siyu Chen, Yaqian Qu, Zhiqiang Hu, Jingquan Sun

**Affiliations:** 1 Institute of Sports Science, Sichuan University, Chengdu, People’s Republic of China; 2 School of Physical Education and Sports, Sichuan University, Chengdu, People’s Republic of China; 3 Department of Physical Education, Sichuan University, No. 24 South Section 1, Yihuan Road, Chengdu, 610065 China

**Keywords:** Endurance training, Lactate, cAMP, Intramuscular triglyceride, Skeletal muscle, Mitochondrial content

## Abstract

The athlete's paradox phenomenon involves the accumulation of intramuscular triglycerides (IMTG) in both insulin-resistant and insulin-sensitive endurance athletes. Nevertheless, a complete understanding of this phenomenon is yet to be achieved. Recent research indicates that lactate, a common byproduct of physical activity, may increase the accumulation of IMTG in skeletal muscle. This is achieved through the activation of G protein-coupled receptor 81 (GPR81) leads to the suppression of the cyclic adenosine monophosphate–protein kinase A (cAMP–PKA) pathway. The mechanism accountable for the increase in mitochondrial content in skeletal muscle triggered by lactate remains incomprehensible. Based on current research, our objective is to explore the role of the GPR81-inhibited cAMP–PKA pathway in the aggregation of IMTG and the increase in mitochondrial content as a result of prolonged exercise. The GPR81–cAMP–PKA-signaling pathway regulates the buildup of IMTG caused by extended periods of endurance training (ET). This is likely due to a decrease in proteins related to fat breakdown and an increase in proteins responsible for fat production. It is possible that the GPR81–cAMP–PKA pathway does not contribute to the long-term increase in mitochondrial biogenesis and content, which is induced by chronic ET. Additional investigation is required to explore the possible hindrance of the mitochondrial biogenesis and content process during physical activity by the GPR81–cAMP–PKA signal.

## Introduction

The body's glucose and lipid metabolism relies heavily on skeletal muscle, which is considered a vital organ, and the accumulation of lipids in muscle away from their normal location can lead to insulin resistance by triggering cytosolic kinase cascades that interfere with molecular insulin signaling [30]. However, endurance athletes have elevated levels of IMTG, which are associated with increased sensitivity to insulin. This phenomenon is sometimes referred to as the athlete's paradox [25]. This suggests that how muscle manages lipid storage is more important than the actual lipid content. However, the molecular mechanisms responsible for this phenomenon require further investigation.

Carbohydrates and lipids serve as the primary sources of fuel used in the mitochondria to sustain muscle contraction during exercise [3]. IMTG plays an important role as the primary fuel source for skeletal muscle energy throughout submaximal exercise [26]. High-intensity interval training (HIIT) can result in metabolic adaptations of skeletal muscle, which include IMTG accumulation and mitochondrial content increase. Data collected in our lab have validated this point: Chen and Zhou et al. found that a 5-week HIIT regimen increased the content of IMTG [4, 5]. Our published results also suggest that a 6-week HIIT program can induce IMTG accumulation and elevate the amount of mitochondria in skeletal muscle [34]. However, a large amount of literature has reported that endurance exercise is the optimal exercise protocol to induce IMTG accumulation [11, 15, 33]. Therefore, to examine the GPR81 function in regulating the IMTG content accumulation during training, we finally chose to adopt the exercise model with endurance exercise training.

Recently, the many functions of lactate in controlling skeletal muscle performance have gained prominence, and it has been presented that exercise has a positive influence on skeletal muscle performance by means of lactate. Zhou et al. performed an experiment using intramuscular injection of lactate at a concentration of 0.25 M to simulate exercise-induced lactate accumulation. They found that a 5-week injection of lactate increased the level of IMTG and promoted mitochondrial biogenesis in gastrocnemius [43]. Siyu et al. also conducted a chronic injection experiment using lactate and forskolin (an activator of the cAMP). The study discovered that the cAMP–PKA pathway regulated the inhibition of lipolysis, leading to lactate-induced accumulation of IMTG. Lactate injection might also increase mitochondria content, while the contributions of the cAMP–PKA pathway might be limited [4, 5].

Taken together, training can promote skeletal muscle adaptation in IMTG metabolism and mitochondrial content. Lactate, generated during training, might have a similar physiological function. Given this, we speculate that increased intramuscular lactate during ET might regulate the accumulation of IMTG and mitochondrial content. This physiological process could be achieved through the cAMP–PKA pathway. To block the lactate/GPR81 (G-protein-coupled receptor 81) pathway and protect cells from glucose deprivation, we used a putative GPR81 antagonist, 3-hydroxy-butyrate (3-OBA) [37]. Chen et al. found that 3-OBA treatment could inhibit glycolysis and suppress lactate metabolism [4, 5]. Therefore, to verify our hypothesis, we administered intramuscular injections of 3-OBA before each exercise, expecting to examine the GPR81–cAMP–PKA pathway implications on changes in IMTG and Mitochondrial content induced by endurance exercise.

## Materials and methods

### Ethical approval

All applicable institutional and governmental regulations for the ethical use of animals were followed, and the protocols received permission from the Ethics Committee of Sichuan University (No. K2020004).

### Animals

The experiment was conducted using male Wistar rats that were 6 weeks and purchased from Chengdu DaShuo Biological Technology Co., Ltd., China. The rats were kept on a conventional rodent chow diet and had unlimited access to water. They were exposed to 12 h of light and 12 h of darkness each day. All the rats have adapted to the new environment for 1 week.

**Fig. 1:**
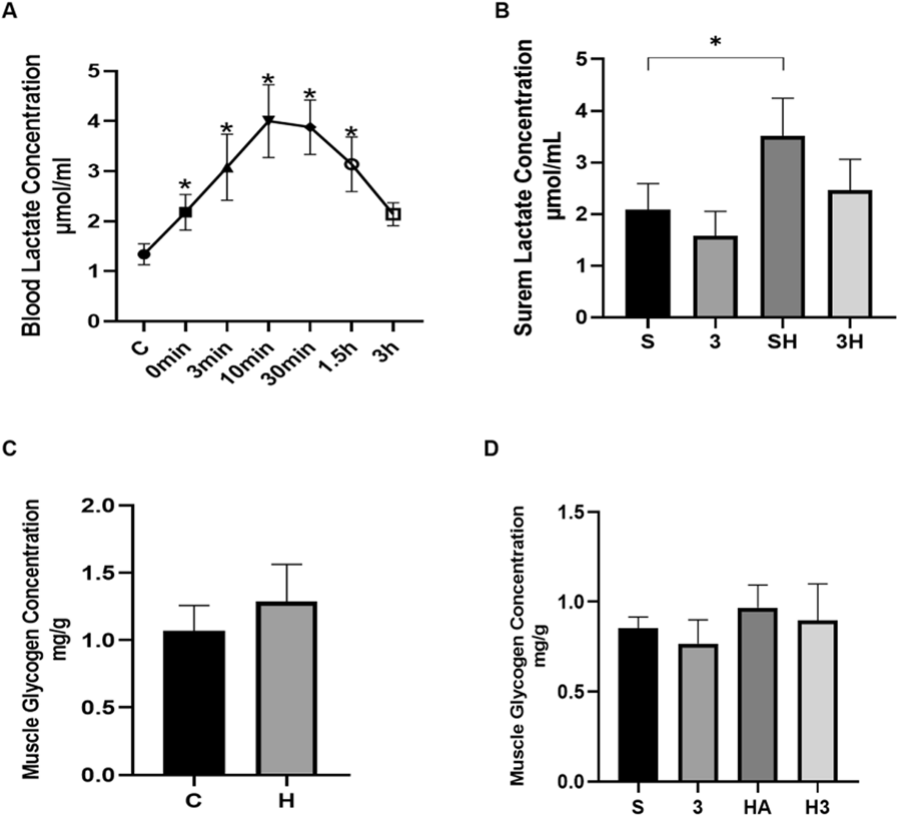
Change of lactate and intramuscular glycogen concentration. **A** Blood lactate concentration of rats was determined at the quiet status and 0 min, 3 min, 10 min, 30 min, 1.5 h, and 2 h after the ET protocol. C, quiet control group (*n* = 5). **B** Serum lactate concentration of rats was determined after 6-week 3-OBA and ET intervention (*n* = 10). **C**, **D** Intramuscular glycoge concentration of rats was determined after 6-week 3-OBA and ET intervention (*n* = 10). S, saline-treated group; 3, 3-OBA-treated group; SH, saline and ET-treated group; 3H, 3-OBA and ET-treated group. The significant differences between the C group and other groups (0 min, 3 min, 10 min, 30 min, 1.5 h, 3 h) were analyzed with the independent-samples *t* test, and the four groups (S, 3, SH, and 3H) were analyzed with the two-way ANOVA. *p < 0.05

In the first experiment, the purpose was to determine the changes following acute ET; we randomly chose Wistar rats (*n* = 5) to experiment with blood lactate concentration (only Fig. 1A). In the subsequent chronic experiment, a random assignment was made of the animals into six groups: control group (C, *n* = 10); ET group (H, *n* = 10); saline-injected group (S, *n* = 10); 3-OBA-injected group (3, *n* = 10); saline-injected and ET group (SH, *n* = 10); 3-OBA-injected and ET group (3H, *n* = 10). The acute group corresponds to the chronic group, which is acute control group (aC, *n* = 10); acute ET group (aH, *n* = 10); acute saline-injected group (aS, *n* = 10); acute 3-OBA-injected group (a3, *n* = 10); saline-injected and acute ET group (aSH, *n* = 10); 3-OBA-injected and acute ET group (a3H, *n* = 10). The chronic group underwent a 6-week administration period and underwent a 6-h fasting period before sampling. The animals were anesthetized 72 h after the final injection by intravenous injection of 45 mg/kg Pentobarbital sodium. After anesthetizing the rat and taking blood, a method involving excessive bleeding was employed for euthanasia. A precise incision was made in a major blood vessel, leading to significant blood loss. This method ensured a rapid and painless death. The rat's vital signs were carefully monitored throughout the procedure to confirm the cessation of heartbeat and breathing. Adhering to the standards and regulations established forth by the Ethics Committee of Sichuan University, we have employed suitable methods for the disposal of rat carcasses. No mortality was observed during the entire duration of the experiment.

### Injection protocol

According to the acute results of our experiment, we found that the 0.03 M 3-hydroxy-butyrate (3-OBA, 54965, Sigma) concentration gave the best effect to activate the level of PKA phosphorylation. Based on this concentration, we examined the time effect and determined the optimal time was 15 min. Based on these results, rats received an intramuscular injection of 0.081 g/kg 3-OBA (0.03 M) in their left gastrocnemius muscle prior to exercise training at 15 min. The compound 3-OBA was dissolved in a solution of normal saline prior to being administered by injection. Control rats received an injection of an equivalent amount of saline solution.

### Treadmill exercise protocol

All exercising rats were subjected to 20 min of the treadmill apparatus (Duan Animal Treadmill Co.Ltd, Guangzhou, China) at a speed of 5–10 m/min for three consecutive days to minimize novelty stress. After the acclimatization period, the 6-week endurance exercise protocol was engaged.

We utilized a VO_2max_ test program to evaluate the rats' VO_2max_ weekly, ensuring that the exercise intensity for the upcoming week consistently aligned with our expectations. The protocol was designed according to previously described methods [1]. Concisely, the beginning speed of the treadmill was 5 m/min. The speed was incremented by 5 m/min every 3 min. The experiments were halted whenever the animals reached a point, where they could no longer sustain jogging on the treadmill, and the VO_2max_ was subsequently calculated. Each ET protocol was approximately 50 min and divided into four parts: (1) warm-up: 70% VO_2max_, 7 min; (2) high-intensity: 75% VO_2max_, 3 min × 6; (3) low-intensity: 65% VO_2max_,3 min × 6; and (4) recovery: 70% VO_2max_, 7 min. All acute intervention groups were killed immediately after 50 min of training or 50 min of resting on the treadmill. They had trained 5 days per week, with 2 days of rest. For a complete schematic illustration of the exercise protocol, see Table 1.

**Table 1:** Speed of 6-week ET protocol (m/min)

	1st week	2nd week	3rd week	4th week	5th week	6th week
Warm-up	12.70	17.18	19.41	21.32	19.83	20.42
High intensity	16.30	22.09	24.95	27.41	25.50	26.25
Low-intensity	9.10	12.27	13.86	15.23	14.17	14.58
Recovery	12.70	17.18	19.41	21.32	19.83	20.42

### Determination of blood lactate concentration

The portable electrochemical device Lactate Scout and Sensors were employed to assess blood lactate levels following acute lactate therapy (SensLab EKF, Germany). Several studies have demonstrated this device's accuracy [28]. We utilized a glass beaker to enclose the rat, with its tail protruding through the water outlet. To activate the vein, submerge the tail in warm water at a temperature of 45–50 degrees for a brief period. Afterward, let it dry and proceed to clean it by rubbing it with a cotton ball soaked in alcohol. Cut approximately 0.2–0.3 cm off the tip of the tail. Wipe away the first drop of blood. After 20 min of rest, the rest blood lactate concentration was measured (C-group). Then, to avoid external stimuli for the following test, the rats were subjected to 50 min of ET exercise and placed in the chamber. After exercise, the blood lactate was measured immediately at 0 min, 3 min, 10 min, 30 min, 1.5 h and 3 h.

We first injected pentobarbital sodium into the rats before the experiment. The anesthetized rats were placed on the rat experimental board (supine position), and the limbs were fixed with cotton thread. To prevent injury during recovery and facilitate the experimental operation of the neck and chest, the two upper incisor teeth of the rats were fixed on the experimental board with cotton thread. Cut the abdominal cavity from the midline of the abdomen, expose the abdominal aorta, and draw blood with a syringe. Serum lactate concentration was measured in the 6-week treatment groups using a microtitre plate-based lactate assay kit (Beijing Solarbio Science and Technology Co., Ltd., China). Following the protocol provided with the kit, 100 µL of serum samples from each rat were analyzed.

### Oil Red O staining of gastrocnemius

The gastrocnemius tissue was subjected to Oil Red O staining after a wash with PBS and fixation with 4% paraformaldehyde. Incubation with 60% Oil Red O followed for a duration of 10 min, with redundant oil red O being removed upon completion of staining. An inverted microscope (NIKON Eclipse Ci) at a magnification of times 400 was used to collect images of Oil Red O-stained gastrocnemius tissue.

### Triglycerides (TG) assay of gastrocnemius

The intracellular TG was quantified employing a TG assay kit (GPO–POD; Applygen Technologies Inc., Beijing, China). Following the manufacturer's prescribed procedure, we weighed and lysed each sample of rat gastrocnemius muscle tissue, with a target weight of 50 ± 5 mg while keeping it on ice. Every individual specimen was put onto a 96-well plate with two identical copies and then combined with the A + B solution from the kit. Prior to this, the samples were heated at a temperature of 70 ℃ for a duration of 10 min. The resultant purple colour was quantified employing a spectrometer at a wavelength of 492 nm, following a 15-min incubation at a temperature of 37 ℃, followed by cooling to ambient temperature. The protein concentration of each specimen, as determined by the BCA test, is then employed to standardize the ultimate results.

### Western blot

At the completion of the 6 weeks of training, 72 h after the final treatment injection and training, all rats were sacrificed. Following the slaughter of the animals, the left gastrocnemius muscle tissue was promptly extracted on ice and then preserved at a temperature of – 80 ℃. The process of extracting total protein from fresh frozen tissue specimens was conducted utilizing the following techniques: we performed Western blot analysis using a pooled sample obtained from the gastrocnemius muscles of 10 rats in each group. Each sample was prepared by combining the tissue lysates from all 10 rats. Using the combined samples, we can reduce the individual variability and provide the accuracy of the results. The mixed gastrocnemius specimens from each group were homogenized in ice-cold RIPA buffer, with roughly 80 mg employed for each sample. Subsequently, they were then subjected to centrifugation at 12000 RPM for 30 min at a temperature of 4 ℃. Protein concentrations were measured by BCA assay (Thermo): a 96-well plate with a clear bottom was loaded with supernatant and gradient diluted protein standards (All samples were measured in duplicate) and then combined with the A + B solution of the kit. The sample is incubated at a temperature of 37 ℃ in a water bath for a duration of 30 min. Afterward, it is allowed to drop down to the ambient room temperature. The resulting color, which is purple, is then quantified using a spectrometer at a wavelength of 562 nm. Sample levels were plotted against the calibration curve, and the supernatant was ultimately rinsed with PBS in preparation for WB analysis.

In summary, a quantity of 20–30 μg of protein was separated on a 10% or 12% SDS–PAGE gel and then transferred to the PVDF membrane. Next, the specimens were obstructed employing 5% skimmed milk for a period of 30–60 min. Antibodies employed for WB were Anti-Rabbit Secondary Antibody (S0001, Affinity), Anti-Mouse Secondary Antibody (S0002, Affinity), GAPDH (AF7021, Affinity), β-tubulin (T0023, Affinity), P-PKA (Ser 99, AF1942, Beyotime), PKA (AF7748, Affinity), P-CREB (Ser 133, MA5-11192, Invitrogen), CREB (9197S, CST), P-HSL (S853, BM4506, Boster), HSL (ABS131896, Absin), P-ATGL (S406, AB135093, Abcam), ATGL (AB109251, Abcam), CPT1b (PA5-79065, Invitrogen), SREBP1c (AF4728, Affinity), FAS (C20G5, CST), PPARγ (AF6284, Affinity), PGC-1α (AB188102, Abcam), COX IV (3E11, CST), SDHA (ab137040, Abcam). Blots were created employing Western Lightning ECL (Affinity). Image J was employed to evaluate all the bands. Protein was normalized to the levels of β-tubulin, GAPDH, or non-phosphorylated corresponding proteins.

### Citrate synthase and complex IV activity assay of gastrocnemius

Mitochondrial citrate synthase (CS) and complex IV (COX IV) activity were determined using the citrate synthase kit and complex IV kit (Beijing Solarbio Science and Technology Co., Ltd., China). Approximately 100 ± 5 mg of mixed gastrocnemius muscle from rats of every group was measured, lysed on ice, and then tested following the protocol provided by the kit. The final values are normalized to the protein concentration of the respective sample measured by the BCA assay.

### Determination of intramuscular glycoge concentration

After obtaining skeletal muscle samples, they were ground in a 1:10 homogenate medium. The resulting mixture was centrifuged at 3000–4000 rpm for 10 min, and the supernatant was collected for testing. In the blank tube, 1 mL of double-steamed water was added, while the standard tube received 1 mL of standard liquid. The test tube was filled with 0.9 mL double-steamed water and 0.1 mL glycogen detection liquid. All tubes were then filled with 2 mL of color-developing solution, mixed, and boiled in water for 5 min. After cooling and additional mixing, the OD value of each tube was measured at a wavelength of 620 nm. Finally, the muscle glycogen content was determined by calculating the ratio to the protein content.

### Statistical analysis

Data were presented as the mean ± standard deviation, and the statistical analyses were conducted using GraphPad Prism software (version 8.0; GraphPad Software, Inc., La Jolla, CA, USA). Independent samples *t* test was used to compare the means of the two groups. Comparisons between multiple groups were performed using the two-way analysis of variance (AVONA). *P* value < 0.05 was considered a statistically significant difference.

## Results

### The time-dependent implications of acute endurance exercise protocol on blood lactate concentration

Figure 1A displays the blood lactate concentration in rats after undergoing an acute ET protocol. Prior to the acute ET, the rats had an average resting blood lactate content of 1.35 ± 0.2 mmol/L. Blood lactate level was 2.18 ± 0.4 mmol/L immediately after exercise (0 min), subsequently increased to 3.08 ± 0.7 mmol/L (3 min) and reached a peak value of 4.00 ± 0.7 mmol/L after 10 min. After reaching its highest point, lactate level gradually decreased to 3.88 ± 0.5 mmol/L (30 min), 3.14 ± 0.5 mmol/L (1.5 h), and 2.14 ± 0.2 mmol/L (3 h). In addition to the 3 h, all of which were statistically significant (*p* < 0.05). These outcomes manifest that this endurance exercise regime effectively elevates lactate levels.

### The effects of 6-week ET intervention and 3-OBA injection on serum lactate concentration and intramuscular glycogen concentration

Contrasted with the quiet control group, the 6-week ET significantly increased the serum lactate concentration (*P* = 0.0238), while the 3-OBA injection slightly decreased the serum lactate concentration (Fig. 1B). In addition, the administration of 3-OBA before each ET intervention effectively blocked the chronic ET-induced increase in serum lactate concentration. These results suggested that chronic ET could increase serum lactate concentration, and 3-OBA injection could suppress the chronic ET-induced elevation in serum lactate concentration. There was no significant increase in intramuscular glycogen after 6 weeks of ET, and no significant change in intramuscular glycogen after 3-OBA intervention (Fig. 1C, D).

### The abundance of intramuscular TG and Oil Red O staining

**Fig. 2:**
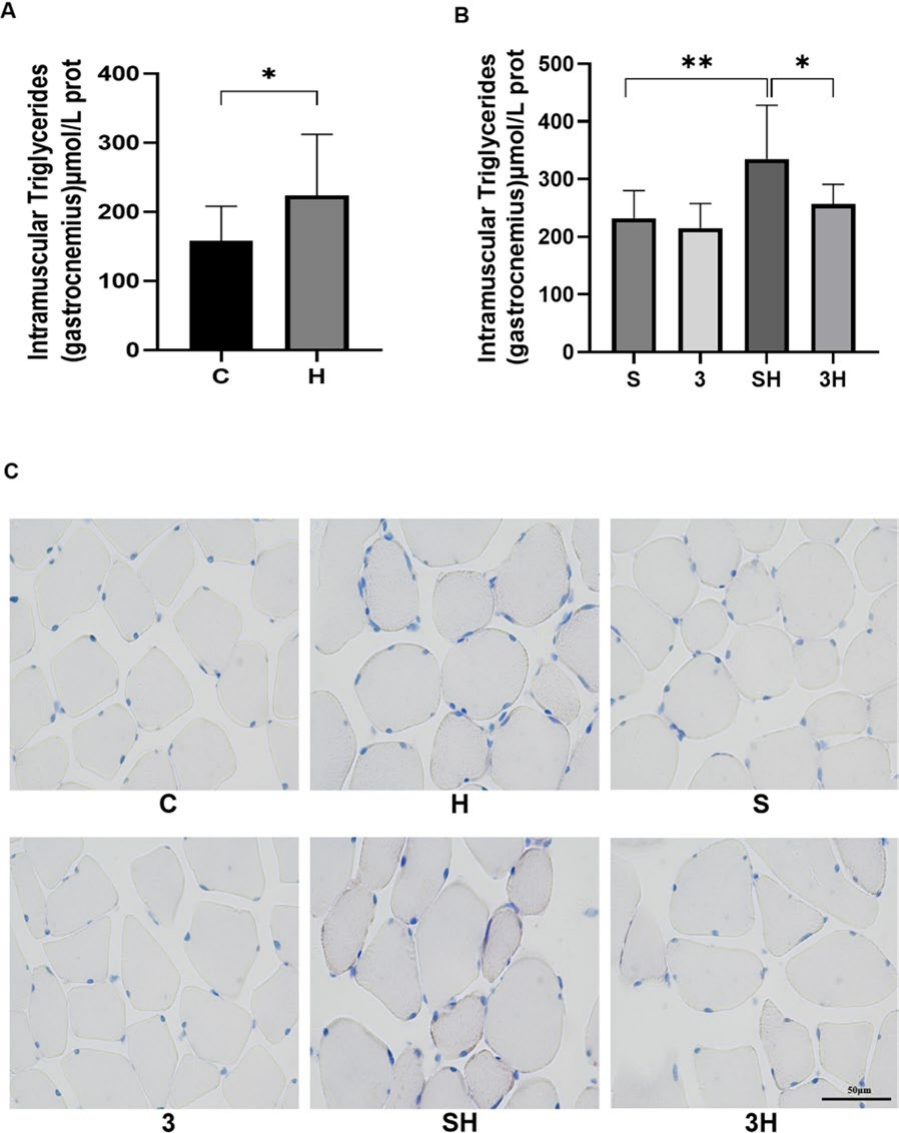
Intramuscular triglyceride variation after 6-week 3-OBA and ET intervention. **A**, **B** IMTG content in gastrocnemius muscle (*n* = 10). **C** Staining of rat gastrocnemius muscle with Oil Red O. **C**, control group; H, ET group; S, saline-treated group; 3, 3-OBA-treated group; SH, saline and ET-treated group; 3H, 3-OBA and ET-treated group. Measurement data were presented as the mean ± SD. The significance of the difference of the two groups **C** and **H** was calculated with the independent samples *t* test, and the four groups (S, 3, SH, and 3H) were analyzed with the two-way ANOVA. *p < 0.05 and **p < 0.01

Contrasted with the quiet control group, 6 weeks of ET significantly enhanced the amount of IMTG in the gastrocnemius muscle of rats (*P* = 0.0417) (Fig. 2A). The content of IMTG after 6-week 3-OBA injection did not change obviously (Fig. 2B). However, the administration of 3-OBA before each ET intervention effectively blocked the chronic ET-induced accumulation of IMTG (*P* = 0.0139) (Fig. 2B). Meanwhile, Staining with Oil Red O showed the same pattern variability as the change in IMTG content (Fig. 2C). Group H exhibited a higher number of micronuclei and more TGs (indicated by a redder color) compared to Group C. The changes observed in Groups S and SH were consistent with those in Groups H and C, respectively. However, Group 3H showed a significantly lower number of myonuclei and TGs compared to Group SH.

### The Effects of acute ET Intervention and 3-OBA Injection on Intramuscular cAMP–PKA pathway

**Fig. 3:**
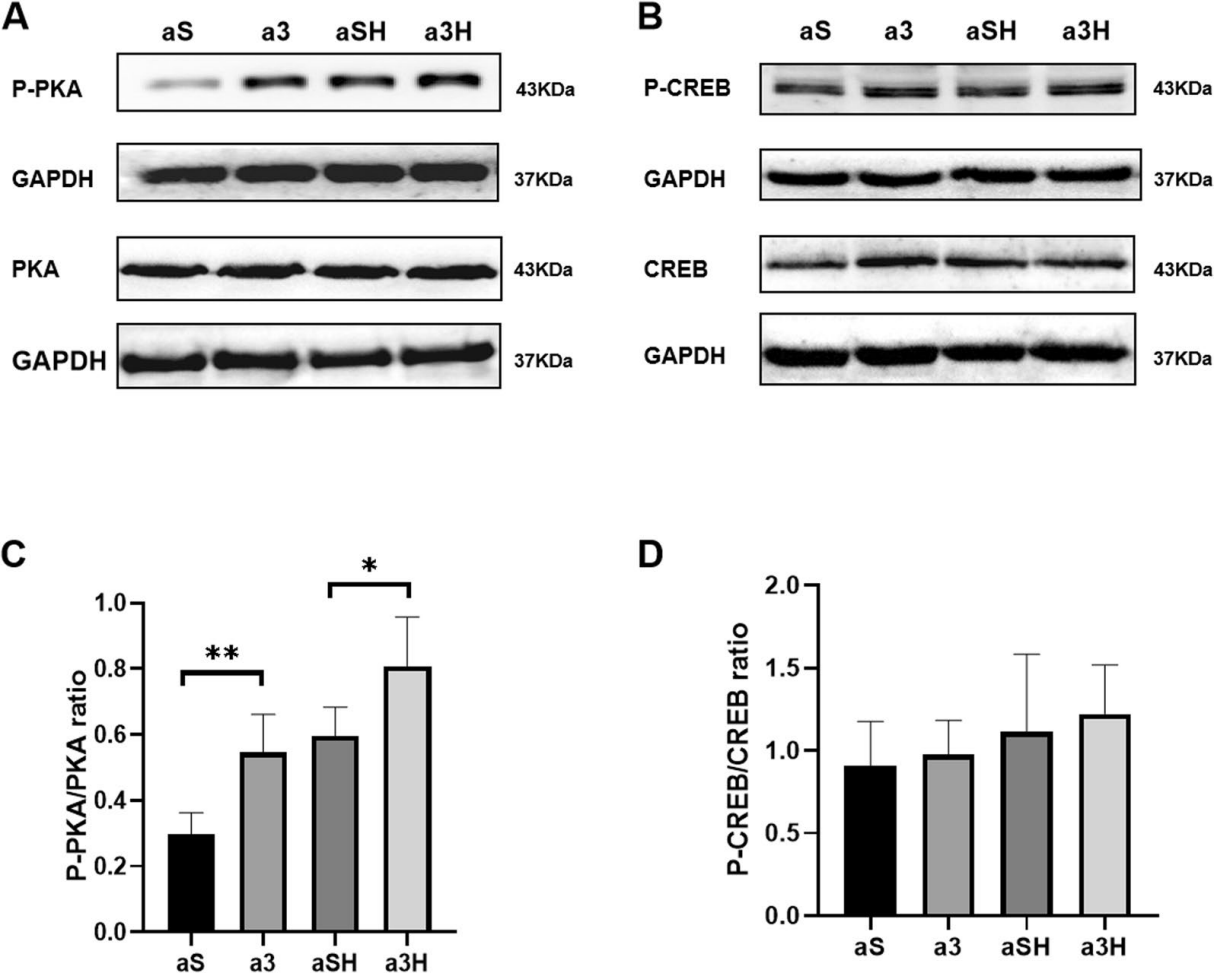
Western blot (WB) analysis of the ratios of P-PKA/PKA and P-CREB/CREB following acute 3-OBA and ET intervention. **A**, **B** WB analysis of P-PKA, PKA, P-CREB and CREB. **C**, **D** P-PKA/PKA ratio and P-CREB/CREB. Three bands per group of rats are used. Relative levels were standardized to GAPDH or β-tubulin. Measurement data were presented as the mean ± SD. The four acute groups (aS, a3, aSH, and a3H) were analyzed with the two-way ANOVA. *p < 0.05. The number of biological repeats (*n* = 10)

The 3-OBA injection increase in P-PKA/PKA (*P* = 0.0381) induced by acute ET (Fig. 3A, C). Nevertheless, there were no significant alterations in the ratio of P-CREB/CREB in the aS, a3, aSH, and a3H groups (Fig. 3B, D).

### The Effects of 6-week ET Intervention and 3-OBA Injection on Intramuscular cAMP–PKA pathway

**Fig. 4:**
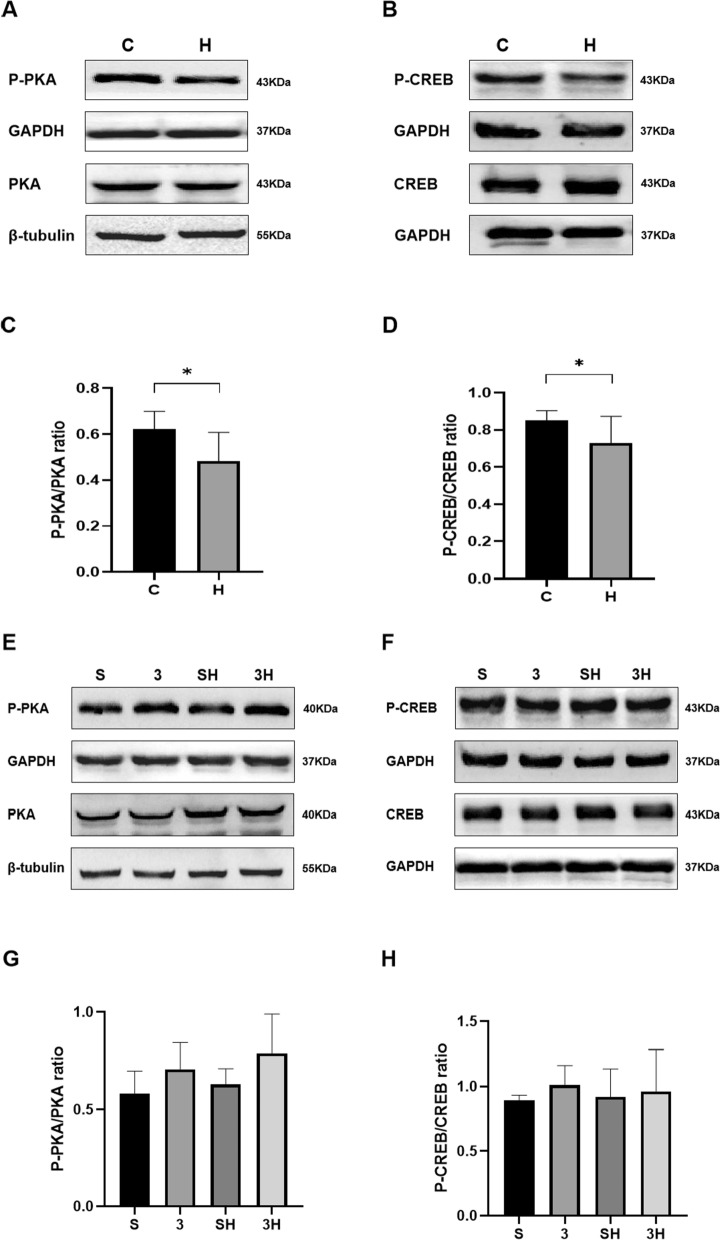
Western blot (WB) analysis of the ratios of P-PKA/PKA and P-CREB/CREB following 6-week 3-OBA and ET intervention. **A**, **E** WB analysis of P-PKA and PKA. **B**, **F** WB analysis of P-CREB and CREB. **C**, **G** P-PKA/PKA ratio. **D**, **H** Ratio of P-CREB/CREB. Three bands per group of rats are used. Relative levels were standardized to GAPDH or β-tubulin. Measurement data were presented as the mean ± SD. The significance of the difference of the two groups **C** and **H** was calculated with the independent samples *t* test, and the four groups (S, 3, SH, and 3H) were analyzed with the two-way ANOVA. *p < 0.05. The number of biological repeats (*n* = 10)

The 6-week ET resulted in a decrease in the intramuscular ratios of P-PKA/PKA (*P* = 0.0178) and P-CREB/CREB (*P* = 0.0282) in the H group compared to the C group (Fig. 4A–D). Meanwhile, the 3-OBA injection blocked the decrease in P-PKA/PKA (*P* = 0.1070) induced by chronic ET (Fig. 4E, G). Nevertheless, there were no significant alterations in the ratio of P-CREB/CREB (*P* = 0.9711) in the S, 3, SH, and 3H groups (Fig. 4F, H).

### The protein of lipolysis-linked proteins P-HSL and HSL, P-ATGL and ATGL, and CPT1b

**Fig. 5:**
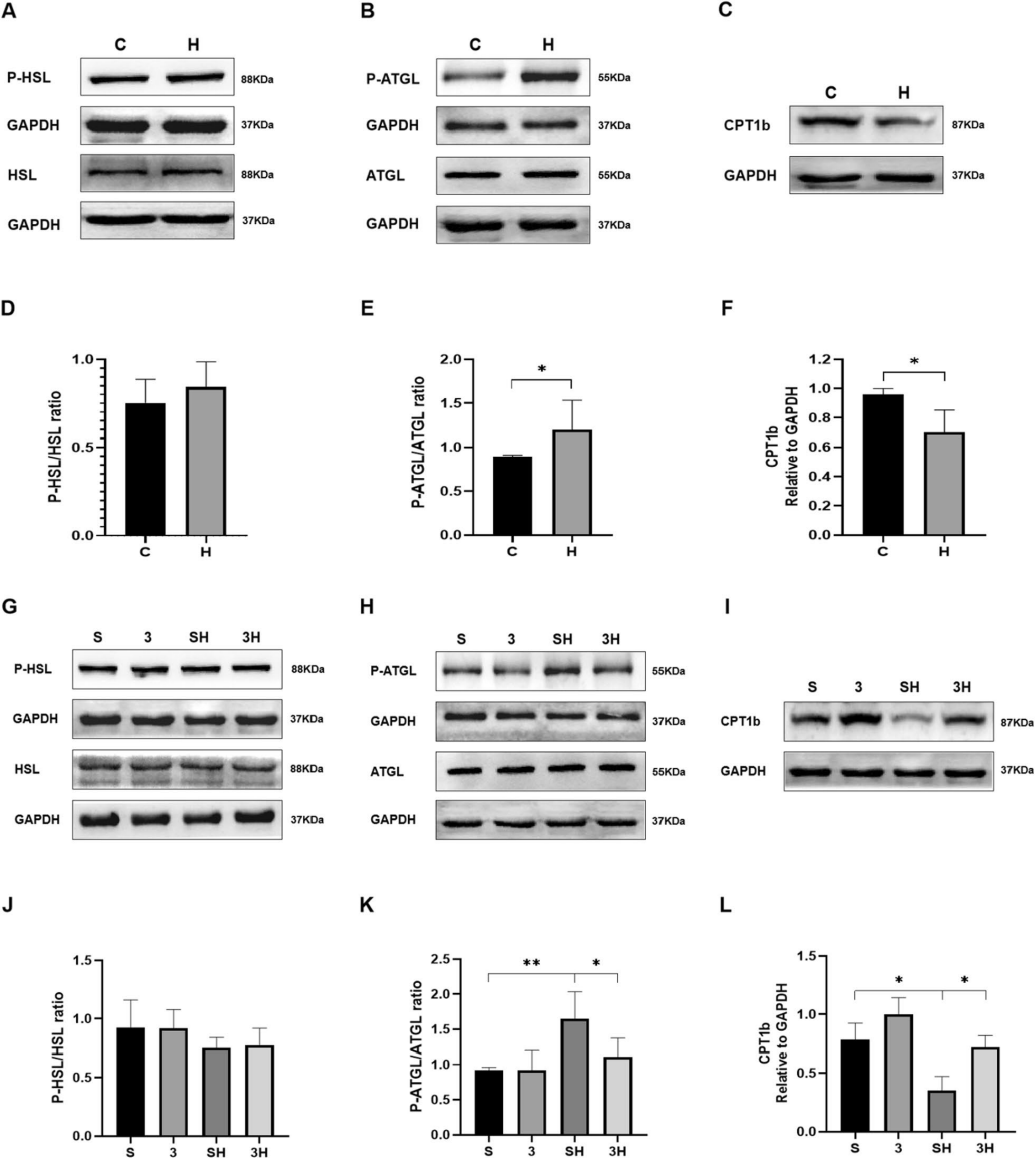
Levels of proteins related to lipolysis after 6 weeks of 3-OBA and ET intervention. **A**, **G** Western blot (WB) analysis of P-HSL and HSL. **B**, **H** WB analysis of P-ATGL and ATGL. (**C**, **I**) WB analysis of CPT1b. **D**, **J** Ratio of P-HSL/HSL. **E**, **K** P-ATGL/ATGL ratio. **F**, **L** Fold protein of CPT1b. Three bands per group of rats are used. Relative levels were standardized to GAPDH. Measurement data were presented as the mean ± SD. The significance of the difference between the two groups **C** and **H** was calculated with the independent samples *t* test, and the four groups (S, 3, SH, and 3H) were analyzed with the two-way ANOVA. *p < 0.05 and **p < 0.01. The number of biological repeats (*n* = 10)

The lipolysis-linked protein levels (P-HSL/HSL, P-ATGL/ATGL, and CPT1b) were used to reflect lipolytic capacity. The 6-week ET intervention did not significantly affect the P-HSL/HSL ratio (*P* = 0.3561). However, it significantly enhanced the phosphorylation of ATGL (*P* = 0.0366) and suppressed the protein of CPT1b (*P* = 0.0469) (Fig. 5A–F). Meanwhile, when the 6-week ET was preceded by a 3-OBA injection, there were no significant differences observed in the P-HSL/HSL ratio (*P* = 0.9700). Moreover, the 3-OBA injection effectively prevented the decrease in CPT1b (*P* = 0.0304) and the increase in P-ATGL/ATGL (*P* = 0.0300) induced by exercise (Fig. 5G–L).

### The protein of lipogenesis-linked proteins SREBP-1c, FAS, and PPARγ

**Fig. 6:**
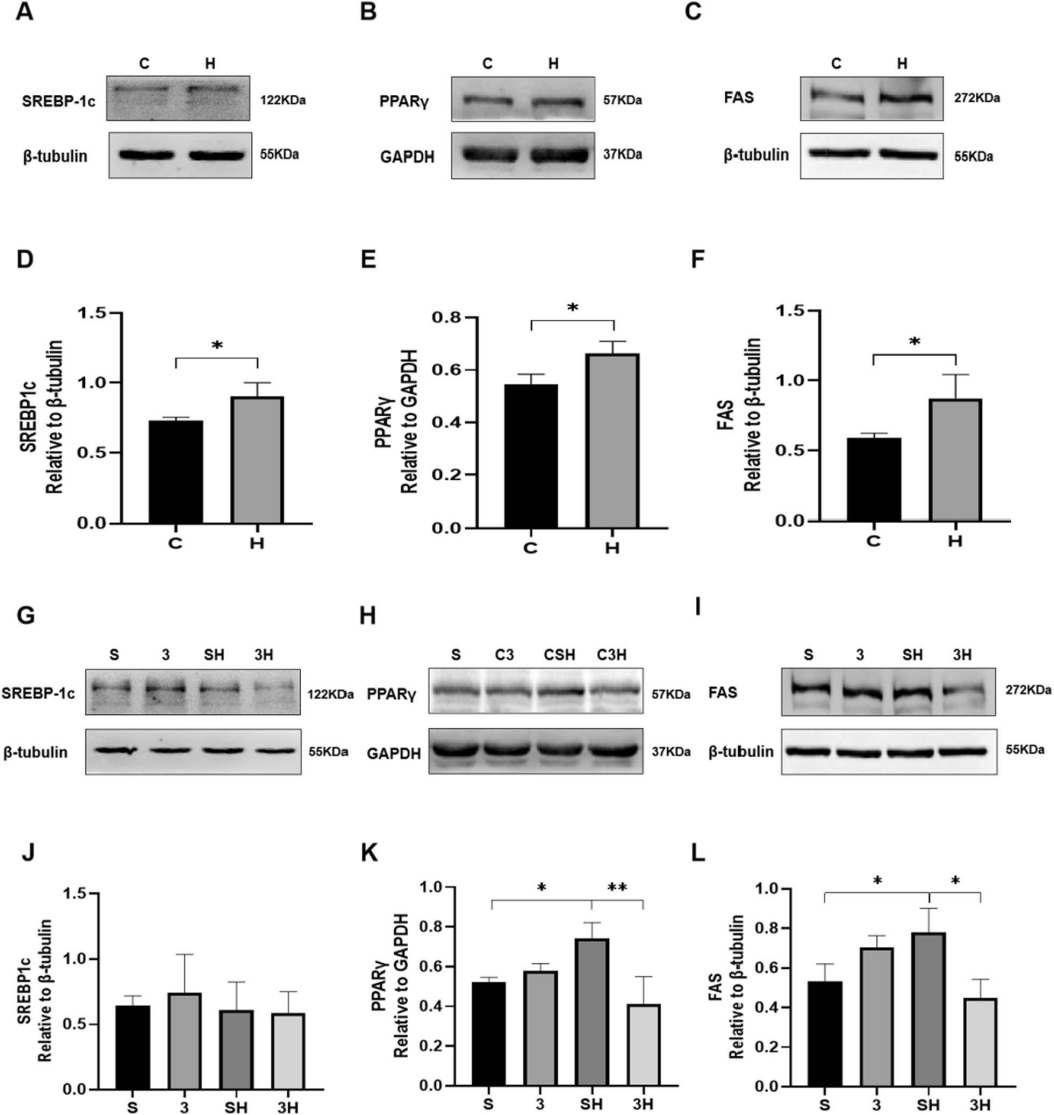
Levels of proteins related to lipogenesis after 6 weeks of 3-OBA and ET intervention. **A**, **G** Western blot (WB) analysis of SREBP-1c. **B**, **H** WB analysis of PPARγ. **C**, **I** WB analysis of FAS. **D**, **J** protein of SREBP-1c. **E**, **K** protein of PPARγ. **F**, **L** Fold protein of FAS. Three bands per group of rats are used. Relative levels were standardized to GAPDH or β-tubulin. Measurement data were presented as the mean ± SD. The significance of the difference between the two groups **C** and **H** was calculated with the independent samples *t* test, and the four groups (S, 3, SH, and 3H) were analyzed with the two-way ANOVA. *p < 0.05 and **p < 0.01. The number of biological repeats (*n* = 10)

The synthesis of TGs is also regulated by lipogenesis-related proteins. Therefore, we examined the SREBP-1c, PPARγ, and FAS protein levels. The 6-week ET intervention significantly increased the protein levels of SREBP-1c (*P* = 0.0426) and FAS (*P* = 0.0487) (Fig. 6A–F). 3-OBA injection before exercise effectively inhibited the increase in FAS (*P* = 0.0108) and PPARγ (*P* = 0.0052) (Fig. 6H, K, I, L). Nevertheless, no significant alterations in SRBEP-1c were observed (*P* = 0.9993) (Fig. 6G, J).

### The protein of intramuscular mitochondrial biogenesis and mitochondrial content biomarkers

**Fig. 7:**
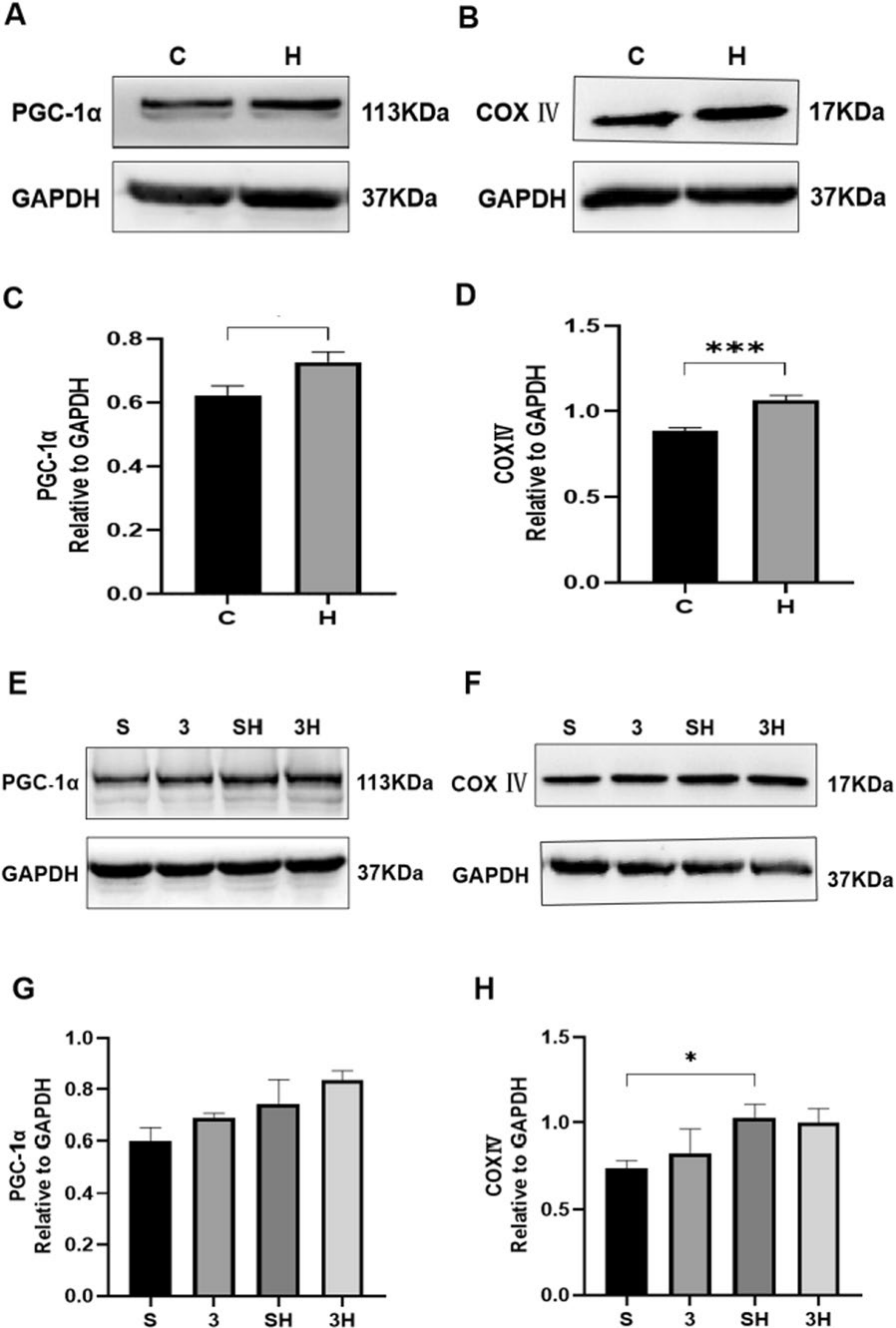
PGC-1α and COX IV protein levels after 6-week 3-OBA and ET intervention. **A**, **E** Western blot (WB) analysis of PGC-1α. **B**, **F** WB analysis of COX IV. **C**, **G** Fold protein of PGC-1α. **D**, **H** Fold protein of COX IV. Three bands per group of rats are used. Relative levels were standardized to GAPDH. Measurement data were presented as the mean ± SD. The significance of the difference between the two groups **C** and **H** was calculated with the independent samples *t* test, and the four groups (S, 3, SH, and 3H) were analyzed with the two-way ANOVA. *p < 0.05 and ***p < 0.001. The number of biological repeats (*n* = 10)

**Fig. 8:**
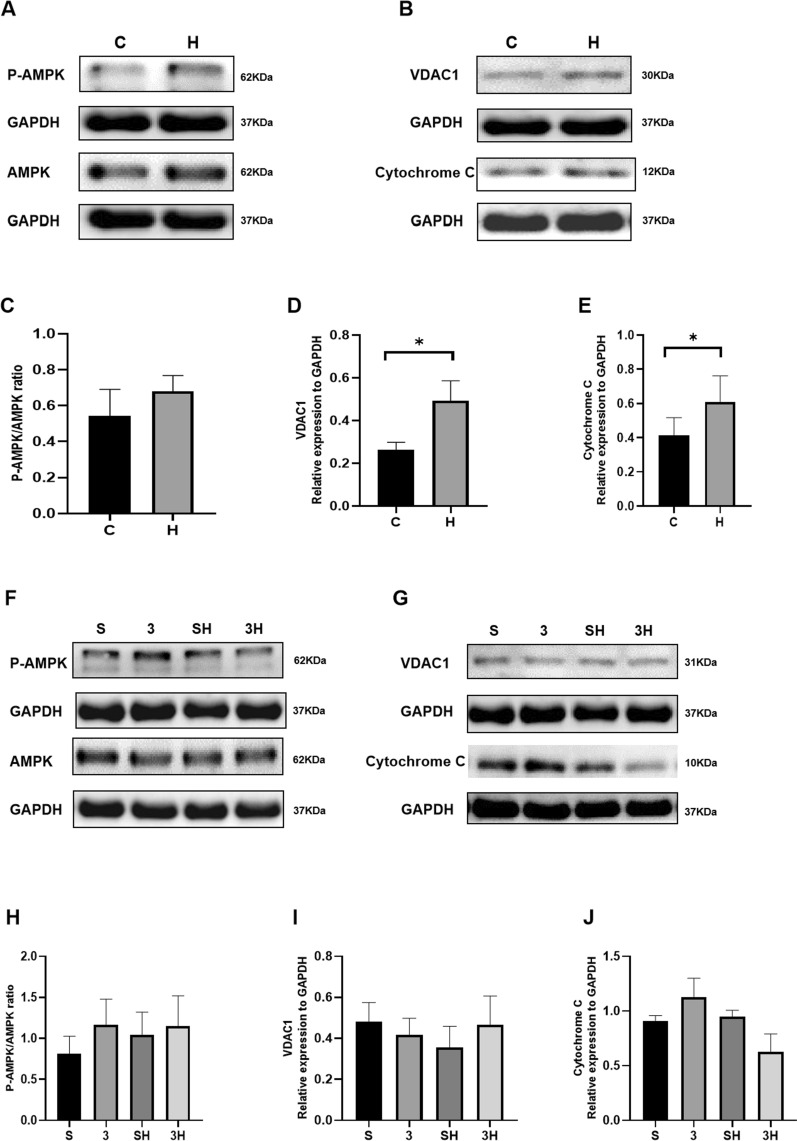
Relative activity levels of AMP-activated protein kinase (AMPK), Voltage Dependent Anion Channel Protein 1 (VDAC1), and Cytochrome C after 6-week 3-OBA and ET intervention. **A**, **B**, **F**, **G** Western blot analysis of P-AMPK/AMPK; VDAC1 and Cytochrome C. **C**–**E**, **H**–**J** Fold protein of P-AMPK/AMPK; VDAC1 and Cytochrome C. Measurement data were presented as the mean ± SD. The significance of the difference between the two groups **C** and **H** was calculated with the independent samples *t* test, and the four groups (S, 3, SH, and 3H) were analyzed with the two-way ANOVA. *p < 0.05; **p < 0.01; and ***p < 0.001. The number of biological repeats (*n* = 10)

To examine the effect of chronic ET on mitochondria and its mechanism, we examined protein levels. As presented in Fig. 7A–D, the PGC-1α (*P* = 0.0145) and COX IV (*P* = 0.0009) protein levels significantly elevated after the 6-week ET intervention. When comparing the SH group and the 3H group, the 3-OBA injection had no significant impact on the protein levels of PGC-1α (*P* = 0.2623) and COX IV (*P* = 0.9879) (Fig. 7E–H). As shown in Fig. 8, compared with group C, group H elevated the protein of VDAC1 (*P* = 0.0171) and Cytochrome C (*P* = 0.0467) after chronic ET.

### The relative activity of mitochondrial content Biomarkers CS and COX IV in gastrocnemius

**Fig. 9:**
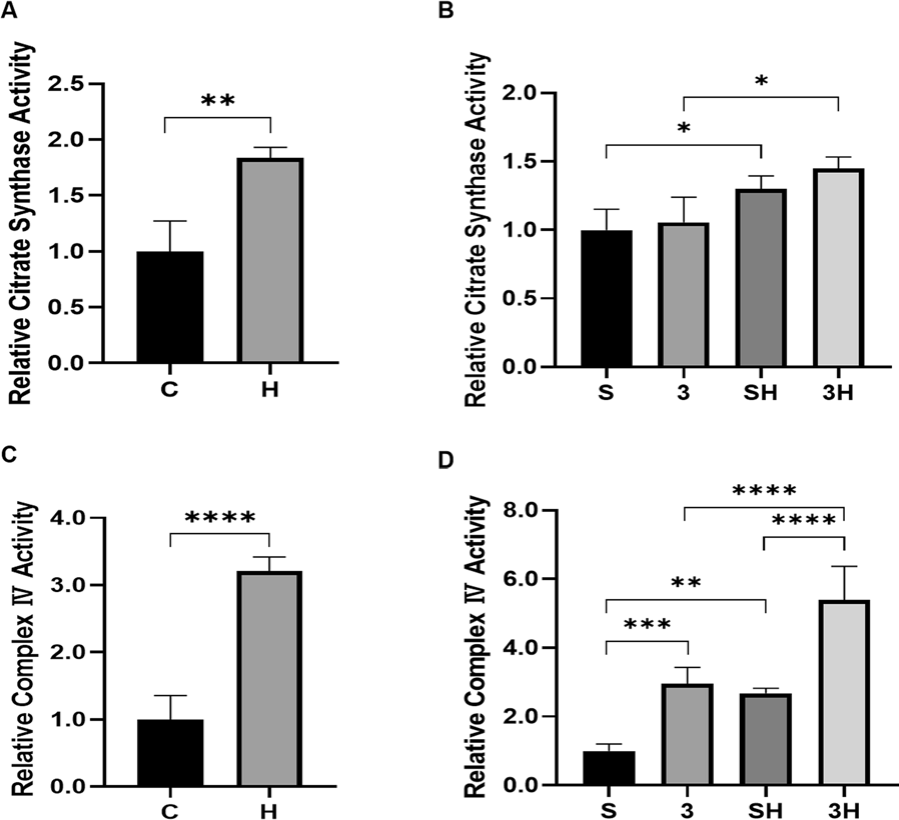
Relative activity levels of citrate synthase (CS) and complex IV (COX IV) after 6-week 3-OBA and ET intervention. **A** Relative activity of CS after 6-week ET intervention. **B** Relative activity of CS after 6-week ET intervention and 3-OBA injection. **C** COX IV relative activity after 6-week ET intervention. **D** COX IV relative activity after 6-week ET intervention and 3-OBA injection. Measurement data were presented as the mean ± SD. The significance of the difference of the two groups **C** and **H** was calculated with the independent samples *t* test, and the four groups (S, 3, SH, and 3H) were analyzed with the two-way ANOVA. *p < 0.05; **p < 0.01; and ***p < 0.001. The number of biological repeats (*n* = 10)

To further verify these results, we assessed the CS and COX IV activity in gastrocnemius mitochondria. The findings were congruent with the alterations reported in the associated proteins. As shown in Fig. 9A–D, the 6-week ET intervention significantly increased the relative activity of CS (*P* = 0.0072) and COX IV (*P* < 0.0001). Furthermore, in Fig. 9D, it can be observed that the injection of 3-OBA further enhanced the exercise-induced increase in COX IV activity (*P* < 0.0001).

## Discussion

Prolonged exercise training is well-known for inducing muscle adaptation in lipid metabolism, which is essential in regulating energy metabolism [35, 39]. As a time-efficient exercise mode, long-term training has been found to induce the accumulation of IMTG and an increase in mitochondrial content [4, 5, 35, 39, 43], which might in part account for the athlete paradox. However, it is noteworthy that athletes who engage in endurance training possess a higher quantity of IMTG [10]. Meanwhile, lactate has a similar effect, inducing IMTG accumulation and increasing mitochondrial content [4, 5, 43]. In this study, we hypothesized that the adaptation in lipid metabolism induced by long-term ET might occur through lactate–GPR81. Based on this, we have conducted a series of experiments and obtained results as follows: (1) chronic ET exercise promoted IMTG accumulation, and this effect was significantly blocked by 3-OBA, (2) IMTG accumulation could be the result of suppressed lipolysis and enhanced lipogenesis, and this suppression of lipolysis and enhanced lipogenesis was inhibited in part by 3-OBA injection; (3) mitochondrial content in skeletal muscle increased after chronic ET and the ET-induced change was further enhanced by 3-OBA injection.

In our experiment, we also found that after an acute ET protocol, the blood lactate concentration in rats was above baseline (1.35 ± 0.2 mmol/L) for 3 h, and the highest concentration achieved was 4.00 ± 0.7 mmol/L. Therefore, data from trained and untrained rodents support the notion that ET effectively increases the lactate level. In addition, the limited effect of 3-OBA injection on intramuscular glycogen by chronic ET has not been reported so far, so it is worth further exploration in the future. Published results from our laboratory suggest that chronic lactate injection has the capacity to induce IMTG accumulation by inhibiting lipolysis and promoting lipogenesis, and it appears to be controlled by the cAMP–PKA pathway [4, 5]. However, whether the GPR81–cAMP–PKA pathway is involved in ET-induced lipid metabolism has not been described.

3-Hydroxy-butyrate (3-OBA) is an inhibitor of GPR81 [38]; however, the question of whether 3-OBA acts as an antagonist of GPR81 is currently under debate. Several studies have provided data showing that 3-OBA neutralizes the implications of extracellular lactate or the well-recognized-specific GPR81 antagonist 3,5-dihydroxybenzoic acid (3,5-DHBA) in many experimental setups [23]. Khatib-Massalha et al. showed that 3-OBA replicates the impacts of GPR81 knockout on the migration of neutrophils to the liver produced by lactate and counteracts the lactate implications in vivo [16]. Finally, 3-OBA has been shown to abolish lactate and 3,5-DHBA-mediated processes in gut stem cell-mediated epithelial growth, recapitulating the impact of GPPR81 knockout [21]. While there is no evidence that 3-OBA interferes directly with the GPR81 receptor or its downstream signal transduction, this partial list of findings indicates that 3-OBA is a functional blocker of GPR81-triggered cellular actions. In our experiment, we found that 6 weeks of 3-OBA injection slightly decreased the serum lactate concentration, and a 6-week ET significantly increased it. More importantly, 3-OBA injection effectively blocked chronic ET-induced increase in serum lactate concentration. It suggested that 3-OBA might inhibit GPR81 based on its ability to decrease blood lactate concentration, and 3-OBA could effectively inhibit chronic ET-induced increase in blood lactate. These results also provided support for our research hypothesis.

We tested the effects of acute ET intervention and 3-OBA injection on intramuscular cAMP–PKA-signaling pathways. Our results suggest that GPR81 inhibits P-PKA/PKA in acute ET, which may be to reduce IMTG consumption and make more use of free fatty acids for energy supply. Because in aerobic endurance exercise, free fatty acids provide the most energy [14, 29]. Next, we performed a 6-week experiment to examine the possible function of the lactate-suppressed cAMP–PKA pathway in chronic ET-induced IMTG acculturation. Consistent with previous studies, we observed that the 6-week ET intervention significantly elevated the abundance of IMTG, and this increase was significantly blocked by 3-OBA injection. It suggests that chronic ET can induce significant IMTG accumulation, and the GPR81–cAMP–PKA pathway might be involved in this process. Meanwhile, the results of the P-PKA/PKA ratios also supported this opinion. However, although the P-CREB/CREB ratio was downregulated after the 6-week ET intervention, no significant alteration was observed among other groups. This is probably due to the fact that CREB is a well-studied transcription factor that is affected by many stimuli, leading to the activation of target genes via transcription [36]. the most core phosphorylation site of CREB is ser133, which is associated with Erk, Ca^2+^, and stress-related-signaling pathways, mainly including kinases, such as CaMK and MAPKAPK2 [27, 38]. Taken together, we conclude that the GPR81–cAMP–PKA pathway may be important in chronic ET-induced IMTG accumulation.

The vast majority of animal adipose tissues are TGs, and Adipose TG lipase (ATGL) and hormone-sensitive lipase (HSL) are the most important TG lipase enzymes [42]. The phosphorylation sites of ATGL are mainly Thr372, Ser406, and Ser430 [22], and we mainly tested Ser406, which catalyzes the first step of TG hydrolysis in muscle after activation and is regulated by PKA [24]. In this paper, we observed that 6-week ET significantly upregulated the ratio of P-ATGL/ATGL, and the change was effectively blocked by 3-OBA injection. This result suggests that chronic ET could induce the phosphorylation of ATGL, and this has the capacity to be controlled by the GPR81–cAMP–PKA pathway. However, the result cannot explain chronic ET-induced IMTG accumulation. Next, we examined another TG hydrolysis enzyme, CPT1b, which is also a key enzyme mediating lipid transportation into mitochondria for fatty acid oxidation (FAO). Specifically, it catalyzes the transfer of long-chain fatty acyl-CoAs from the cytoplasm to the mitochondria via the carnitine shuttle [31]. Surprisingly, we found that the 6-week ET significantly downregulated the protein of CPT1b in the gastrocnemius, and this effect was blocked by 3-OBA injection. It is suggested that chronic ET could inhibit the CPT1b, and the GPR81–cAMP–PKA pathway might be involved. As a key rate-limiting enzyme responsible for regulating triacylglycerol mobilization, we also examined the ratio of P-HSL/HSL but found no obvious change. This is probably due to the fact that HSL, as a hormone-sensitive lipase, could be affected by many influencing factors, such as adrenaline and contractions [9, 17, 18]. Taken together, our results regarding lipolysis-related proteins suggest that although chronic ET can increase the phosphorylation of ATGL to promote the decomposition of IMTG, the attenuation of CPT1b induced by chronic ET might induce impairment of β-oxidation of fatty acids, leading to skeletal muscle lipid accumulation [40]. Meanwhile, the blocking effects of 3-OBA suggest that the GPR81–cAMP–PKA pathway might take part in the changes of ATGL and CPT1b induced by chronic ET.

Correspondingly, lipogenesis-related proteins (SREBP-1c, FAS, and PPARγ) were also examined in this paper. SREBP-1C is one of the major enzymes that regulate fat production [8]. PPARγ is a significant protein involved in the production of fats. It belongs to the nuclear receptor superfamily of transcription factors that are activated by ligands. PPARγ is known as a key regulator of the process of adipocyte differentiation (adipogenesis) [20, 32]. FAS is essential in synthesizing long-chain fatty acids and is regulated by SREBP-1c [7]. In our study, we found that 6-week ET increased the protein of SREBP-1c, PPARγ, and FAS, which might explain why chronic ET could induce IMTG accumulation. However, in the subsequent blocking experiment, we found that only the elevation of PPARγ and FAS could be inhibited by 3-OBA injection, there are no distinct change trends in SREBP-1c. The findings on lipogenesis-related proteins above indicated that the GPR81–cAMP–PKA pathway may be involved in chronic ET-induced IMTG accumulation by upregulating PPARγ and FAS.

It is noteworthy, however, that exercise-induced intramuscular lipid accumulation is not an isolated physiological change, which is often accompanied by enhanced catabolic capability [12]. At the same time, long-term exercise has been proven to induce adaptations of mitochondrial biogenesis and mitochondrial content [2, 13, 41]. The mechanism remains unclear. Prior research indicates that while lactate injections can enhance mitochondrial levels, their impact appears to be restricted [4, 5]. For further verification, we examined related indicators of mitochondrial biogenesis and mitochondrial content to examine the importance of the GPR81–cAMP–PKA pathway in chronic adaptation of mitochondrial function induced by ET. PGC-1α regulates lipid metabolism by regulating the citric acid cycle and fatty acid oxidation [6]. Cytochrome c oxidase (also called mitochondria complex IV, COX IV) is validated as a surrogate of mitochondrial content in human skeletal muscle [19]. Based on our findings, following a 6-week ET intervention, the levels of PGC-1α and COX IV exhibited significant increases. However, it was observed that the levels of PGC-1α and COX IV protein did not show further enhancement after the injection of 3-OBA. Nevertheless, the activities of CS and COX IV were notably enhanced after the 3-OBA injection. It indicated that chronic exercise could increase mitochondrial biogenesis and mitochondrial content by upregulating PGC-1α and COX IV. However, the GPR81–cAMP–PKA pathway does not appear to facilitate this chronic exercise adaptation; in contrast, the signaling pathway might have an inhibitory function. To further verify our results, we examined the activity of CS (Citrate synthase) and COX IV in the gastrocnemius. The results showed that 6-week ET obviously enhanced the activity of CS and COX IV, and 3-OBA injection further augmented the increase in COX IV activity. 6 weeks of ET significantly enhance the protein of VDAC1 and Cytochrome C. However, it did not cause associated changes in VDAC1 and Cytochrome C after 3-OBA injection. Moreover, although the changes in P-AMPK/AMPK were not significant, the rise of P-AMPK and AMPK, respectively, may induce the activation of PGC-1α-signaling pathway, and it should further investigate whether GPR81 activates PGC-1α through AMPK-signaling pathway to enhance mitochondrial function.

Taken together, our outcomes suggest that long-term ET can enhance the protein of PGC-1α and COX IV and the CS and COX IV activity. However, the GPR81–cAMP–PKA-signaling axis appears to have negative effects, which might slightly offset the positive effects of lactate on mitochondria content reported in previous studies [4, 5]. This suggests that there might be other, more important, lactate-mediated mechanisms that modulate mitochondria content. Additional research is required to validate this idea and explore the existence of other important signaling pathways in the future.

We primarily propose to determine the accumulation of IMTG, a common occurrence among endurance athletes. Given recent evidence demonstrating that lactate, a well-known exercise metabolite, can enhance IMTG accumulation by inhibiting the cAMP–PKA-signaling pathway through binding to GPR81, we further examined whether IMTG accumulation is mediated by lactate using 3-OBA, a GPR81 inhibitor. Based on our findings, we find that lactate–GPR81 induces the accumulation of IMTG and restrains mitochondrial activity through modulation of the cAMP–PKA-signaling pathway without obviously affecting changes in mitochondrial content. Consequently, we propose that long-term endurance exercise in athletes repeatedly elevates lactate levels, leading to the inhibition of the intramuscular cAMP–PKA-signaling pathway and facilitating the accumulation of IMTG. This process benefits athletes by increasing their energy reserves. However, it is important to note that lactate does not promote mitochondrial function and activity. This suggests that exercise has other significant mechanisms for enhancing mitochondrial function and activity. During ET, skeletal muscle utilizes stored IMTG as a fuel source to meet the energy demands of exercise, which results in the conservation of glycogen consumption. This study is primarily focused on elucidating the impact of GPR81 on IMTG accumulation during long-term exercise, which is distinct from the IMTG accumulation induced by a high-fat diet. Although a high-fat diet also stimulates the biogenesis of mitochondria in skeletal muscle, the composition of the accumulated lipids varies, particularly for molecular species containing palmitoleate. Considering the robust oxidation capacity of IMTG in aerobic metabolism and its ability to provide a sustained and stable energy supply, we conclude that lactate plays a favorable role in promoting IMTG accumulation among endurance athletes. However, it has a negative impact on mitochondrial function and activity.

## Limitation

Our study only suggested that the lactate–GPR81-suppressed cAMP–PKA-signaling axis probably counter-regulated the mitochondria content and did not further explore the other possible mechanisms. This study could benefit from additional experiments following PKA inhibition, which would further substantiate the regulatory function of GPR81 in the cAMP–PKA pathway.

## Conclusions

Based on our findings, we speculate that the GPR81–cAMP–PKA pathway has the capacity to contribute to long-term ET-induced IMTG accumulation. Furthermore, chronic ET could increase mitochondrial biogenesis and mitochondrial content, and the GPR81–cAMP–PKA pathway might have a reverse effect on this adaptation. Therefore, future studies are warranted to confirm our findings and to investigate the potential mechanism of how lactate increases mitochondria content.

## Data Availability

All data generated or analyzed during this study are included in this published article.
